# Nucleic acid amphiphiles: Synthesis, properties, and applications

**DOI:** 10.1016/j.omtn.2023.05.022

**Published:** 2023-06-03

**Authors:** Amu Gubu, Xueli Zhang, Aiping Lu, Baoting Zhang, Yuan Ma, Ge Zhang

**Affiliations:** 1 Law Sau Fai Institute for Advancing Translational Medicine in Bone & Joint Diseases, School of Chinese Medicine, Hong Kong Baptist University, Hong Kong SAR, China; 2 Aptacure Therapeutics Limited, Kowloon, Hong Kong SAR, China; 3 State Key Laboratory of Natural and Biomimetic Drugs, School of Pharmaceutical Sciences and Chemical Biology Center, Peking University, No. 38 Xueyuan Road, Beijing, China; 4 Institute of Integrated Bioinfomedicine and Translational Science, School of Chinese Medicine, Hong Kong Baptist University, Kowloon Tsai, Hong Kong 999077, China; 5 Institute of Precision Medicine and Innovative Drug Discovery, HKBU Institute for Research and Continuing Education, Shenzhen 518000, China; 6 School of Chinese Medicine, Faculty of Medicine, The Chinese University of Hong Kong, Hong Kong SAR, China

**Keywords:** MT: Oligonucleotides: Therapies and Applications, nucleic acid amphiphile, oligonucleotide, aptamer, self-assembly, liposome, cell membrane

## Abstract

Nucleic acid amphiphiles, referring to nucleic acids modified with large hydrophobic groups, have been widely used in programmable bioengineering. Since nucleic acids are intrinsically hydrophilic, the hydrophobic groups endow nucleic acid amphiphiles with unique properties, such as self-assembling, interactions with artificial or biological membranes, and transmembrane transport. Importantly, the hybridization or target binding capability of oligonucleotide itself supplies nucleic acid amphiphiles with excellent programmability. As a result, this type of molecule has attracted considerable attention in academic studies and has enormous potential for further applications. For a comprehensive understanding of nucleic acid amphiphiles, we review the reported research on nucleic acid amphiphiles from their molecular design to final applications, in which we summarize the synthetic strategies for nucleic acid amphiphiles and draw much attention to their unique properties in different contexts. Finally, a summary of the applications of nucleic acid amphiphiles in drug development, bioengineering, and bioanalysis are critically discussed.

## Graphical abstract

**Figure:**
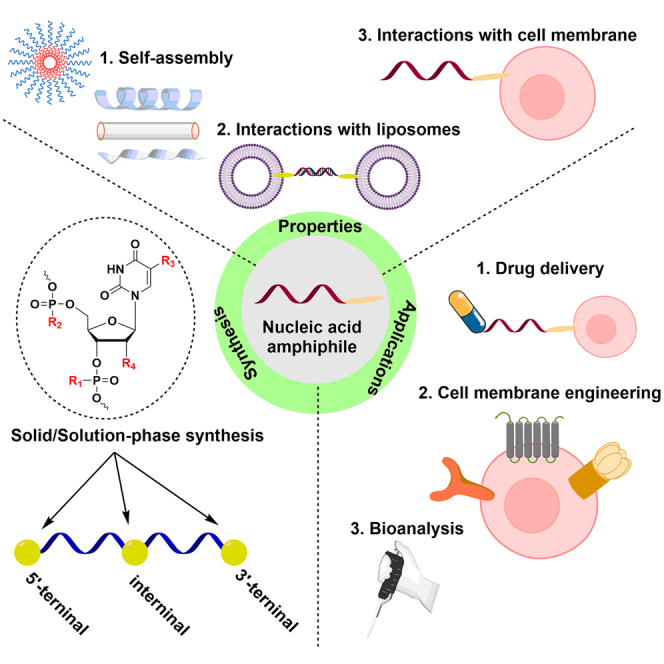

## Teaser

Ma and colleagues review the development on nucleic acid amphiphiles referring to nucleic acids modified with large hydrophobic groups. They summarize the synthetic strategies, highlight the unique properties (self-assembly, interactions with membranes, and transmembrane transport), and delve deeper into the applications (drug development, bioengineering, and bioanalysis).

## Introduction

Nucleic acids, including deoxyribonucleic acid (DNA) and ribonucleic acid (RNA), are biomacromolecules that play extremely important roles in cells. The biological functions of nucleic acids include carrying genetic information (genomic DNA and mRNA), regulating gene expression (microRNA, siRNA, and lncRNA), and biocatalysis (ribozymes). With the development of nucleic acid synthesis methods, nucleic acid-based nanotechnology, therapies and diagnostic tools have accomplished impressive achievements over the last few years.^1^^,^^2^^,^^3^ For example, nucleic acid-based nanostructures have been applied in drug delivery.^4^ siRNA and antisense oligonucleotides have emerged as effective gene therapies with several approved drugs,^5^ and aptamers have shown their potential both in therapies and diagnosis.^6^ Despite their excellent biocompatibility and high efficiency, native nucleic acids face challenges when applied in a biological context. For instance, it is difficult for native nucleic acids to be transported across negatively charged cell membranes owing to their hydrophilicity and negatively charged phosphate groups. Moreover, nucleases inside and outside cells might degrade nucleic acids and reduce their efficiency. Chemical functionalization of nucleic acids can improve their properties including nuclease resistance, delivery efficiency, pharmacokinetic properties, and pharmacodynamic properties. Among various chemical modifications, nucleic acids conjugated with hydrophobic moieties, which result in nucleic acid amphiphiles, have attracted intensive attention owing to their unique properties.

Nucleic acid amphiphiles with increased hydrophobicity have high affinity toward the cellular membrane and unique self-assembly properties. Thus, nucleic acid amphiphiles have proven to be an effective strategy for nucleic acid drug delivery and building blocks for constructing nanostructures that have applications in biological contexts. In this review, we first thoroughly discuss the synthesis methods of nucleic acid amphiphiles by comparing the pros and cons of solid-phase and liquid-phase coupling methods and review recent progress in synthesis method development. Next, the unique properties of nucleic acid amphiphiles, including self-assembly and interactions with membranes are discussed in more detail. Finally, the applications of nucleic acid amphiphiles in drug delivery, cell membrane engineering, and bioanalysis are critically discussed for diagnosis and treatment.

## Synthetic methods of nucleic acid amphiphiles

**Figure 1:**
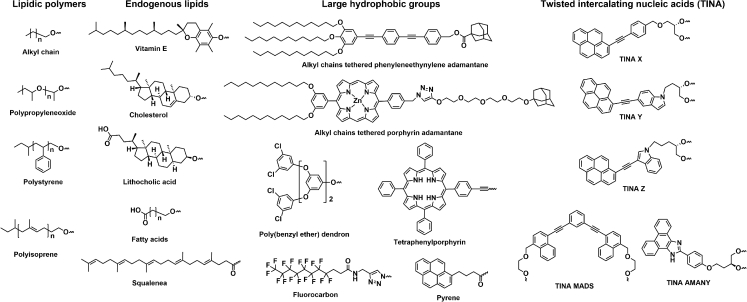
Representative hydrophobic moieties that were utilized to prepare nucleic acid amphiphiles The hydrophobic moieties are roughly divided into four groups: lipidic polymers, endogenous lipids, large hydrophobic groups, and twisted intercalating nucleic acids (TINAs).

Nucleic acids are usually synthesized using an automated DNA/RNA synthesizer with phosphoramidite chemistry on solid supports (controlled pore glasses). The phosphoramidites derived from protected nucleosides are coupled through a four-step reaction cycle to assemble the nucleic acid chain. After chain assembly, the nucleic acid chains are cleaved from the solid supports and deprotected to obtain the final product. During this process, various hydrophobic moieties such as lipidic polymers, endogenous lipids, large hydrophobic groups, and DNA intercalators can be attached to the nucleic acids, resulting in nucleic acid amphiphiles (Figure 1). The synthesis methods of nucleic acid amphiphiles can be roughly divided into solid-phase and solution-phase modification methods. In solid-phase modification methods, the hydrophobic moieties are introduced before the nucleic acid chains are cleaved from the solid supports. In solution-phase modification methods, the hydrophobic groups are introduced after the nucleic acids are hydrolyzed from the solid supports and deprotected. The solid-phase and solution-phase modification method each have their own set of limitations and advantages, and are complementary in certain applications. In the following section, we summarize the chemistry used in nucleic acid amphiphile synthesis and highlight recent advances in solution-phase modification methods.

### Solid-phase synthesis of nucleic acid amphiphiles

Based on the conjugation position of hydrophobic moieties on nucleic acids, nucleic acid amphiphiles can be classified into three categories: 5′ terminal-functionalized, 3′ terminal-functionalized, and internal-functionalized nucleic acid amphiphiles. The phosphoramidite chemistry-based solid-phase synthesis method is commonly used for nucleic acid amphiphiles, which enables the synthesis of all oligonucleotide/oligodeoxynucleotide variants (3′ terminal, 5′ terminal, and internal). However, the synthesis strategies vary depending on the conjugated hydrophobic moieties. In this review, we discuss the primary hydrophobic modifications utilized in solid-phase synthesis.

#### Synthesis of 5′ terminal-functionalized nucleic acid amphiphiles

Because the synthetic direction of the solid-phase DNA/RNA synthesis is from the 3′ terminal to the 5′ terminal, 5′ terminal modification of hydrophobic groups is easily accomplished through a standard DNA/RNA synthesis procedure using hydrophobic group phosphoramidites (Figure S1A). To date, hydrophobic moieties conjugated at the 5′ terminus of oligonucleotides using phosphoramidite chemistry include polystyrene,^7^ poly(benzyl ether) dendron,^8^ naphthenic acids,^9^^,^^10^^,^^11^^,^^12^ vitamin E,^13^ and cholesterol.^14^ Furthermore, by rationally tailoring the structure of phosphoramidites, multiple hydrophobic moieties can be introduced to the 5′ terminus of nucleic acids to produce optimized properties,^12^ such as photo-responsive siRNA using photo-responsive chemical linkers (that is, o-nitro-benzeneethanol) to connect siRNA and hydrophobic groups (that is, cholesterol and vitamin E).^15^^,^^16^ In addition to phosphoramidite chemistry, researchers have also explored the potential of other chemistries in the synthesis of 5′ terminal-functionalized nucleic acid amphiphiles. Shea et al. covalently attached phospholipid to the 5′ terminal of antisense oligonucleotides via H-phosphonate chemistry and applied them to antiviral activities (Figure S1B).^17^ Guzaev and Manoharan took advantage of a substitution reaction between thiol and 2-chloroacetamide and successfully synthesized long-chain alkane and cholesterol-modified oligonucleotides in high yield (Figure S1C).^18^ In addition, the amide formation reaction (Figure S1D),^19^^,^^20^ copper-catalyzed alkyne-azide cycloaddition (Figure S1E),^21^^,^^22^ and Sonogashira coupling (Figure S1F)^23^^,^^24^ have also been applied for the solid-phase synthesis of nucleic acid amphiphiles. Among of them, H-phosphonate chemistry and the substitution reaction between thiol and 2-chloroacetamide have not been widely adopted owing to the instability of reaction intermediates and the relatively harsh reaction conditions. By contrast, phosphoramidite chemistry is widely used owing to its high efficiency and modularity. However, synthesis of the corresponding phosphoramidite requires expertise in nucleic acid chemistry, which limits its further application. Copper-catalyzed alkyne-azide cycloaddition, the amide formation reaction, and Sonogashira coupling are also widely used owing to their satisfactory yields, ease of operation, and availability of functionalized oligonucleotides. It is worth noting that reaction conditions must be fine-tuned to gain ideal yields.

#### Synthesis of 3′ terminal-functionalized nucleic acid amphiphiles

Although it is easier to conjugate hydrophobic moieties at the 5′ terminal of nucleic acids, conjugation to the 3′ terminus is also necessary in circumstances. For nucleic acid drug development, two significant challenges are circulatory stability *in vivo* and cell delivery efficiency. Although conjugating a nucleic acid drug with a hydrophobic group can improve the delivery efficiency of nucleic acid drugs, it is crucial to determine the conjugated locations of hydrophobic groups. For instance, 3′ terminal modifications of siRNAs significantly enhance the resistance to 3′ exonucleases, which are highly expressed in serum and intracellular conditions.^25^ Conversely, 5′ terminal modifications of siRNAs significantly reduce RNAi activity.^26^^,^^27^ Therefore, 3′ terminal modifications are attractive alternative strategies that can conserve siRNA activity with improved drug delivery efficiency and serum stability. For other biological applications, 5′ and/or 3′ terminal modification can provide various modules to construct more complex structures for specific functions. Therefore, researchers have also put extensive efforts into investigating the coupling of hydrophobic groups to the 3′ terminal. The key to the synthesis of 3′ terminal-functionalized nucleic acid amphiphiles is acquiring hydrophobic group-modified solid supports. Solid supports modified with hydrophobic groups can be obtained by coupling these groups to the solid supports’ extending linkers (strategy 1, Figure S2A), or adding hydrophobic group-conjugated linkers to the solid supports (strategy 2, Figure S2B), followed by a standard DNA/RNA synthesis procedure to obtain the target nucleic acid amphiphiles. In strategy 1, the modified oligonucleotides have larger residual groups, which can negatively affect the function of the final product. By contrast, in strategy 2, hydrophobic group-conjugated linkers have a less-redundant structure. Collectively, researchers can flexibly apply an alternative strategy depending on the availability of solid phases or the desired level of product quality.

#### Synthesis of internal-functionalized nucleic acid amphiphiles

Internal-functionalized nucleic acid amphiphiles can be obtained by incorporating hydrophobic group-modified phosphoramidites. Hydrophobic moieties can be modified on nucleotides either at the 2′ position of the sugar ring^28^^,^^29^ (Figure S3A), nucleobases^30^ (Figure S3B), or non-nucleotide phosphoramidites (TINAs in Figure 1 and structures in Figure S3C).^31^^,^^32^^,^^33^^,^^34^ Various chemistries can be applied for the synthesis of internal-functionalized nucleic acid amphiphiles by coupling the hydrophobic moieties to the active groups (for example, alkyne group and iodine modification) in the nucleoside/non-nucleoside. It is considerably easier to obtain internal-functionalized nucleic acid amphiphiles, because they pose fewer challenges in phosphoramidite synthesis. Importantly, the steric hindrance of hydrophobic group-modified phosphoramidites may severely reduce the coupling efficiency during solid-phase synthesis, resulting in low yields. Hence, internal-functionalized nucleic acid amphiphiles are widely prepared by coupling the hydrophobic moieties to the active groups.^35^

The solid-phase modification method for nucleic acid amphiphile synthesis is advantageous for its convenience, high efficacy, and high yield. However, the use of robust deprotection conditions (usually ammonium hydroxide, methylamine, or mixtures of the two) hinders its utility in synthesizing ester/halogen-based nucleic acid amphiphiles. In addition, limited scalability in synthesis (particularly in a pilot scale) and high reagent consumption further constrain its application.^36^ Therefore, introducing hydrophobic moieties after nucleic acids are cleaved from solid supports and deprotected is a promising complementary method for large-scale nucleic acid amphiphile synthesis. This type of method is referred to as a solution-phase modification method for nucleic acid amphiphile synthesis, as described in the next subsection.

### Solution-phase synthesis of nucleic acid amphiphiles

**Figure 2:**
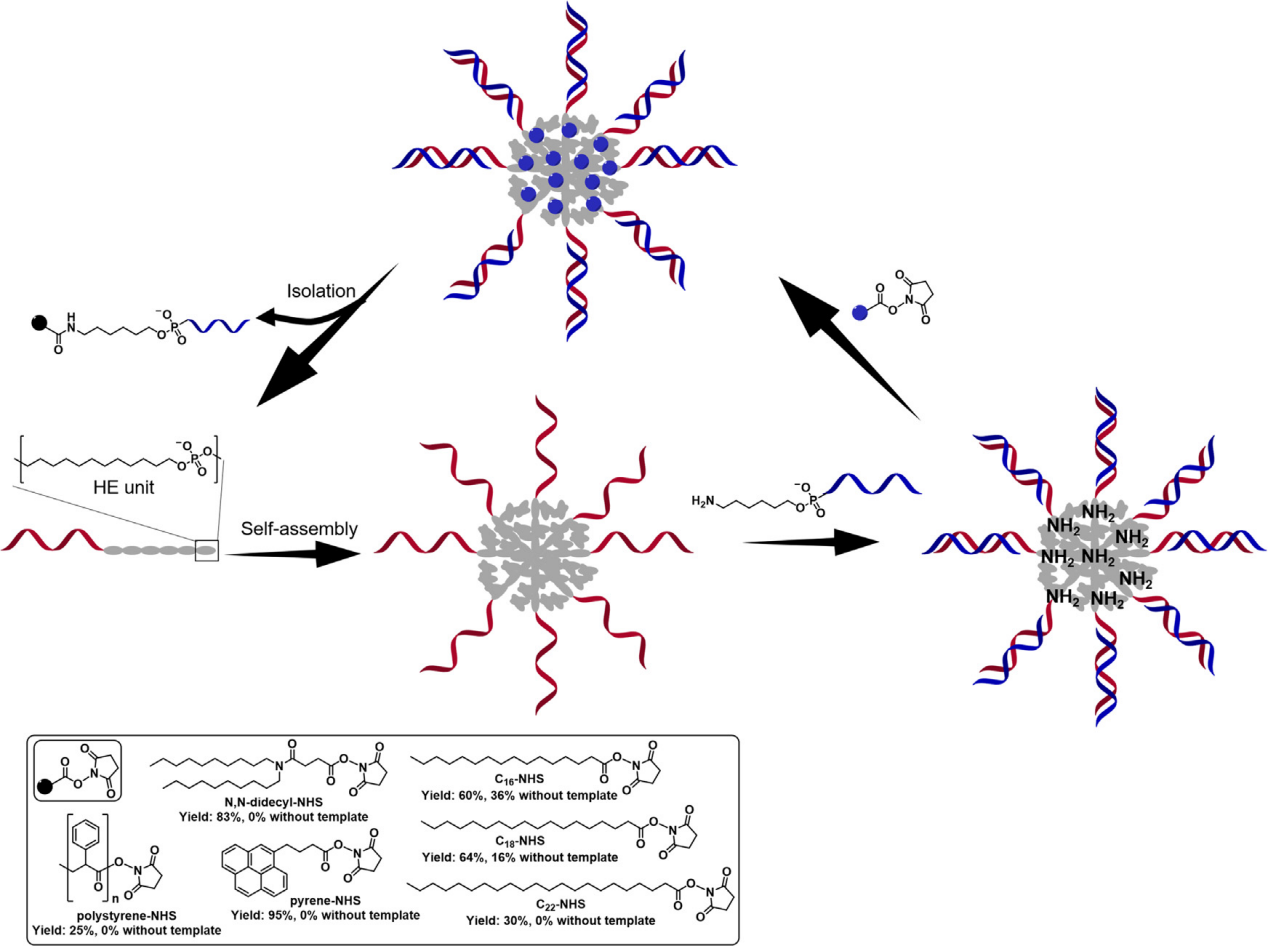
Synthetic methodology for DNA micelles as nanoreactors which efficiently functionalize DNA with hydrophobic organic molecules

Because of solvent incompatibility between hydrophobic moieties and hydrophilic nucleic acids, the preparation of nucleic acid amphiphiles in solution is relatively challenging. Raouane et al. applied maleimide-sulfhydryl chemistry^37^ for the in-solution synthesis of squalene-modified siRNA. The solvents and other reaction conditions must be carefully selected to obtain a satisfactory yield. An amide formation reaction using HATU (2-(7-azabenzotriazol-1-yl)-N,N,N′,N′-tetramethyluronium hexafluorophosphate) as a coupling agent^38^ has also been developed to conjugate a series of hydrophobic molecules to siRNA^39^ with high yield. However, for most coupling reactions, the low yield arising from solvent incompatibility remains an obstacle to expand the structural diversity of nucleic acid amphiphiles.^12^^,^^40^^,^^41^ Recently, researchers designed elegant strategies to improve yields of solution-phase modification methods for nucleic acid amphiphile synthesis. Trinh et al. reported a micelle-templated method for improving the yields of DNA conjugated with highly hydrophobic groups.^42^ In this method, they regarded DNA micelles that were self-assembled by commercial DNA amphiphiles consisting of six units of hexamethylene as nanoreactors. Then, a complementary, non-hydrophobically modified DNA was hybridized to them, leaving functional amino group toward the micellar core. The added hydrophobic groups with activated N-hydroxysuccinimide esters of behenic acid (C_22_), stearic acid (C_18_), palmitic acid (C_16_), a branched (bis-C_10_) N,N′-didecyl chain, chromophore pyrene, and preformed polystyrene could condense in the micellar core owing to hydrophobic interactions. An increased local concentration of reactive groups in the micellar core significantly improved the conjugation yields (Figure 2). Pearce et al. reported a surfactant aided method for nucleic acid amphiphile formation. In this strategy, ionic surfactant cetyl trimethylammonium bromide electrostatically adhered to the phosphate groups of nucleic acids enabled the complexes to be dissolved in organic solvents. A transformation yield higher than 80% was obtained.^43^ Subsequently, Liu et al. expanded the coupling types and substrates of this strategy.^44^ Apart from covalent conjugation, researchers have also explored non-covalent approaches for the synthesis of supramolecular nucleic acid amphiphiles. Albert firstly synthesized supramolecular nucleic acid amphiphiles using the host-guest reaction between β-cyclodextrin and amantadine.^45^ A coupling efficiency higher than 70% was obtained with any hydrophobic moiety of interest and the obtained supramolecular nucleic acid amphiphiles formed DNA-decorated vesicles through self-assembly. This method supplies an efficient, time-saving, and universal strategy for nucleic acid amphiphile synthesis. Furthermore, the reversibility of host-guest interactions allowed the construction of stimuli-responsive DNA nanostructures which have application potential in the fields of drug delivery, cancer diagnosis, and so on.^46^^,^^47^ In addition to host-guest interactions, the interactions between nucleic acid intercalating agents and nucleic acids have also been utilized for the construction of amphiphilic cholesterol-modified siRNA.^48^ It should be noted that, while recent covalent methods such as the micelle-templated method and surfactant aided method have succeeded in increasing reaction yields, they are only applicable to the synthesis of terminal-modified nucleic acid amphiphiles. Theoretically, non-covalent methods can be easily used to synthesize internal-modified nucleic acid amphiphiles. However, researchers have only just conducted preliminary efforts in this field. With further exploration of new non-covalent interactions, it is possible that new methods for the synthesis of internal-modified nucleic acid amphiphiles may be developed in the future.

In the following section, we provide a comprehensive overview of the strategies and chemistries used for the synthesis of nucleic acid amphiphiles with varying structures. Nucleic acid amphiphiles represent a unique subclass of modified nucleic acids. In the past decades, numerous new chemistries have been discovered and employed to expand the chemical diversity of modified nucleic acids. The potentials of these chemistries for the synthesis of nucleic acid amphiphiles remains to be explored in terms of yields and synthesis scales.

## Properties of nucleic acid amphiphiles

Owning to their amphipathicity and programmability, nucleic acid amphiphiles exhibit unique properties in different contexts and thorough studies of these properties are critical for further applications. Nucleic acid amphiphiles can assemble into various nanostructures, and hydrophobic groups endow nucleic acid amphiphiles with the ability to interact with liposomes and cell membranes. Detailed studies on the factors that affect the properties of nucleic acid amphiphiles would enable rational design of nucleic acid amphiphiles for specific applications. In this section, we introduce research focusing on the properties of nucleic acid amphiphiles including self-assembly, nucleic acid amphiphile-mediated liposome assembly/fusion, and interactions with cell membranes. Factors affecting these properties are discussed in detail.

### Self-assembly of nucleic acid amphiphiles

Nucleic acid amphiphiles can self-assemble into various nanostructures driven by hydrophobic interactions. Hydrophobic groups reported for nucleic acid amphiphile construction include lipids,^49^ hydrophobic polymers, molecules with π-conjugated systems,^50^ and dendrons. Spherical micelles are the most common morphology of micelles assembled from nucleic acid amphiphiles conjugating lipids or flexible polymers. The critical micelle concentration (CMC) of nucleic acid amphiphiles, the hybridization ability, and the morphology of the formed nanostructures are critical properties for further applications. Factors affecting these properties include the types and quantities of hydrophobic groups, the position of hydrophobic modifications, the properties of linkers between nucleic acid and hydrophobic groups, the nucleic acid sequences, and the solution environment.

The CMC is an important parameter to evaluate the stability of micelles formed via nucleic acid amphiphiles. It has been reported that the CMC of nucleic acid amphiphiles decreases as the hydrophobicity of hydrophobic moieties increases. For example, Gosse et al. found that the CMC of nucleic acid with chalcone-dihexadecane tags was only 10 nM, which was considerably lower than that of nucleic acid amphiphiles conjugating cholesterol (studies on cholesterol conjugated 10 mers reported either a 150 μM CMC or no CMC at all below 1 mM). Based on theoretical derivations, if we assume that chalcone-dihexadecane is equivalent to a cholesterol molecule plus two C_16_ alkyl chains, the observed difference in CMC should be 2^16^, approximately 65,000.^39^

**Figure 3:**
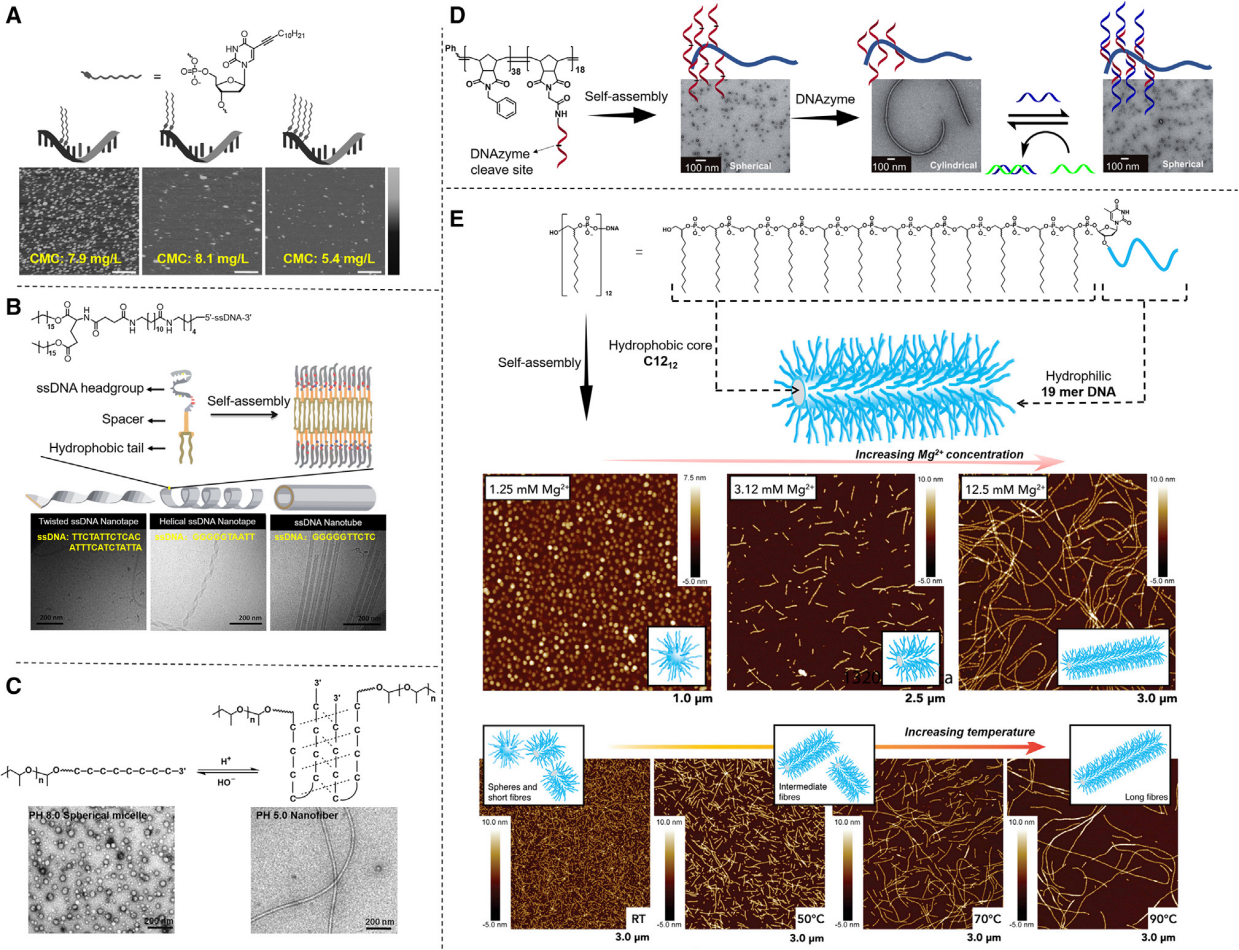
Self-assembly of nucleic acid amphiphiles (A) Atomic force microscope (AFM) height images of single-stranded (ss) lipid-DNA micelles whose CMC changed with different hydrophobic modification sites and modification quantities. Scale bars, 200 nm. The vertical scale is 20 nm (adapted from Anaya et al.^51^). (B) Transmission electron cryomicroscopy (TEM) images of twisted ssDNA nanotapes, helical ssDNA nanotapes, and ssDNA nanotubes formed by nucleic acid amphiphiles with varied ssDNA in different lengths and sequences (adapted from Pearce and Kokkoli^52^). (C) The reversible pH-dependent morphological changes of nucleic acid amphiphiles observed by TEM (adapted from Zhao et al.^53^). (D) TEM images of sequence-dependent assembly of DNA-brush copolymers into micelles with spherical or cylindrical morphologies (adapted from Chien et al.^54^). (E) AFM images of Mg^2+^/temperature-dependent morphological changes of C12_12_-DNA amphiphiles (adapted from Dore et al.^55^).

Similarly, Liu et al. reported that nucleic acid amphiphiles conjugated with diacyllipid have a CMC lower than 10 nM, whereas those conjugated with less hydrophobic monoacyllipid cannot form micelles even at millimolar concentrations.^11^ Anaya et al. investigated the influences of different quantities and positions of lipid molecule modification on CMC (Figure 3A). In this study, the researchers attached dodec-1-yne (C_12_H_22_) to the 5 position of uridine. Then, incorporation of dodecane moieties with varied positions and quantities was allowed by a DNA/RNA synthesizer. As expected, when they increased the amount of modified lipidic dodecanes, the CMC of the resulting conjugates decreased.^51^ Apart from the hydrophobicity of hydrophobic moieties, the configuration and sequences of nucleic acids have also been reported to be correlated with CMC. With the same lipidic chains (biotadecane of triotadecane), Pokholenko et al. demonstrated that nucleic acid amphiphiles with a sequence of 15 mer poly(dA) have a lower CMC than those with 15 mer poly(dT).^12^

The ability to hybridize with complementary strands or recognize target proteins is another parameter for further application of nucleic acid-formed micelles. In most cases, the hybridization capability of nucleic acids is preserved.^7^^,^^8^^,^^56^^,^^57^^,^^58^ For aptamer amphiphiles, spacers between aptamers and hydrophobic groups are critical for the recognition ability. Wu et al. reported that aptamer amphiphiles without spacers or with hydrophilic spacers could form globular micelles while amphiphiles with hydrophobic spacers could form bilayer nanotapes. The formation of micelles improved the binding capability of aptamer amphiphiles with polyethylene glycol (PEG) spacers to their targets.^43^^,^^59^ Waybrant et al. found that aptamer amphiphiles with no spacer (NoSPR) have lower binding ability to their target than native aptamers. Aptamer amphiphiles with spacers such as PEGs (PEG_4_, PEG_8_, and PEG_24_), alkyl (C_12_ and C_24_), or oligonucleotide (T_10_ and T_5_: 10 and 5 thymine; and A_10_: 10 adenine) have higher binding affinity than NoSPR but do not restore affinity to that of the free aptamer.^47^

Many factors affect the morphology of formed nanostructures. While the nucleic acid amphiphile design is important as mentioned above, in regard to the types, quantities, and placement of the hydrophobic moieties across the DNA/RNA strand (terminal, internal, or mixed), the hydrophobic/hydrophilic ratio of the nucleic acid amphiphiles appears to play a vital role in determining the morphology of the formed nanostructures.^50^ With a higher hydrophobic/hydrophilic ratio, which can be achieved by increasing the hydrophobicity of hydrophobic moieties or shortening the length of nucleic acids, the nucleic acid amphiphile tends to form vesicles,^60^^,^^61^ nanotapes,^43^ nanotubes,^52^^,^^62^ and nanowires^8^ instead of spherical micelles (Figure 3B). DNA-π amphiphiles with large π-surfaces as the hydrophobic moieties are also molecule species that tends to form lamellar nanostructures owing to strong π-π stacking forces.^50^ Moreover, the negative charges of the nucleic acid itself further influence the morphology of nucleic acid amphiphiles depending on ionic strength and the pH of the solution.^19^ Regulation of the morphology of nucleic acid amphiphiles has been a research hotspot because of the close relationship between function and morphology.^63^ By tuning the hydrophobic/hydrophilic ratio or changing the solution environment with external stimuli, researchers have achieved the reverse transformation of different morphologies of nanostructure formed by nucleic acid amphiphiles. These studies paved the way for nucleic acid amphiphile-based drug delivery systems. To date, exogenous stimuli that are reported to trigger morphological transformation include pH,^64^ complementary nucleic acids,^54^^,^^65^ enzymes,^54^^,^^60^ and solvents,^66^ which usually alter the hydrophobic/hydrophilic ratio of nucleic acid amphiphiles. Chien et al. prepared DNA brush copolymer amphiphiles by conjugating multiple DNA strands on polymeric hydrophobic chain backbone. These amphiphiles assembled into spherical micelles under native conditions. By adding a longer DNA strand and its complementary strand, they achieved reversible transition between the cylinder and sphere phases (Figure 3D).^54^ Similarly, they successfully achieved transformations between spherical micelles and vesicles using a PEG-DNA as a stimulus.^65^ Not only the length of nucleic acids but also the specific structures may be involved in inducing morphological transformations of micelles. Taking i-motifs as an example, which are four-stranded DNA secondary structures formed via a protonated cytosine (C:C^+^) base pairing in acidic conditions which dissociates into random coils in slightly basic conditions,^67^ Zhao constructed DNA-b-poly(propyleneoxide) amphiphiles that would undergo *in situ* transition between diblock and triblock structures through dissociation and formation of i-motif structures upon pH changes. They established a method of morphology-shifting from spherical micelles to nanofibers (Figure 3C).^53^ Likewise, Yan et al. achieved morphology-shifting from spherical micelles to multi-layer vesicles^64^ via the pH-induced formation of i-motif structures. Albert also transformed the aggregation of DNAsome from spherical micelles into open mesh structures with pH-dependent i-motif structures.^68^ Recently, Zhang et al. reported that cholesterol-DNA amphiphiles assembled into spherical micelles at low pH value and hierarchically changed to one-dimensional nanorods spontaneously by altering the pH.^69^ Divalent ions and temperature can also function as stimuli. Sleiman’s group successfully constructed a DNA amphiphile with a 19-mer DNA tagged with 12 tandem C_12_. Upon increasing the concentration of Mg^2+^, the assembly transformed from micelles into fibers with a concentration-dependent fiber length. Interestingly, the researchers found that the fibers elongated with an increase in temperature, which provided unique thermosetting properties (Figure 3E).^55^ Such novel mechanisms of self-assembly are worthy of further study.

### Interactions between nucleic acid amphiphiles and liposomes

Liposomes are commonly used as models to study interactions between nucleic acid amphiphiles and membranes. Moreover, functionalization of liposomes with nucleic acid amphiphiles has been proven to be a convenient method of constructing liposome-based drug delivery systems and biosensors.^70^ Because nucleic acid-cholesterol conjugates are easy to prepare without the tendency of self-assembly,^39^^,^^71^ many nucleic acid amphiphiles reported to interact with liposomes are nucleic acid-cholesterol conjugates. Owing to the hydrodynamic force generated by the highly charged oligonucleotides, hydrophobic forces alone are not sufficiently strong to ensure strong membrane anchoring by a single cholesterol modification.^72^ Therefore, researchers usually increase the amount of cholesterol to increase the binding strength to the lipid membranes of nucleic acid amphiphiles.^73^^,^^74^ However, there have also been cases in which a single cholesterol modification at the termini of nucleic acids was successfully applied to liposome functionalization and drug delivery, and the observed difference mainly due to the variations in the relative short length of nucleic acid sequence and three-dimensional structure like G4 structure formed by nucleic acid itself, which could decrease ratio of hydrodynamic force in the nucleic acid aphiphiles.^75^ The linker between cholesterol and nucleic acid matters for the interactions between amphiphiles and liposomes. Incorporation of cholesterol molecules will disturb the biolayer structure and dynamics, which introduce significant condensation of membrane lipids. Adding a tetra(ethylene glycol) (TEG) linker between cholesterol and nucleic acid can reduce this effect.^76^ Banchelli et al. studied the effect of the liposome surface coverage of these TEG-linked cholesterol-DNA amphiphiles on the conformation of nucleic acids over phospholipid membranes. The conformation of nucleic acids transited from a quasi-random coil to a relatively rigid state owing to the charge repulsion between negatively charged nucleic acids, and the hybridization rate with the complementary strand became slow. It is worth noting that sufficient ion concentration in the solution is necessary for the insertion of nucleic acid amphiphiles into the membrane structure, especially for liposomes formed by lipid molecules with negatively charged phosphate groups. Aside from the factors mentioned above, the composition of liposomes also has a certain impact on the insertion rate of nucleic acid amphiphiles.^77^ Furthermore, cholesterol has been widely used to anchor nucleic acid nanostructures on membranes. In this application, the amount and orientation of cholesterol impact anchoring, which is more complicated.^78^ In addition to cholesterol, dual lipid molecules,^79^^,^^80^ vitamin E,^81^^,^^82^ and other modifications have also been used in the study of the interactions between nucleic acid amphiphiles and liposomes. Compared with cholesterol modification, dual lipid molecules can be more stably anchored on the phospholipid bilayer membrane. Nakatani’s group designed a novel amphiphilic DNA with nine nucleotide with hydrophobic regions at one end and octyl phosphotriester linkages on the phosphate backbone. Its hydrophobic regions were expected to be inserted into the lipid membrane. Interestingly, such nucleic acid amphiphiles alone cannot be efficiently incorporated into lipid membranes. However, the addition of complementary oligonucleotides to hydrophilic regions improves incorporation efficiency. Researchers thought that single hydrophobic region-oligonucleotides would form micelles themselves, which wrap the hydrophobic regions inside. However, the addition of a complementary strand destroyed the micelle structure, released the hydrophobic regions, and allowed the insertion of hydrophobic groups into the lipid membranes.^83^ This finding indicated the possibility of developing complementary strand responsive methods for liposome labeling. After the hydrophobic regions of nucleic acid amphiphiles are incorporated into liposomes, further properties of liposomes can be predicted and tailored, including nucleic acid-mediated liposome assembly and nucleic acid-mediated liposome fusion.

**Figure 4:**
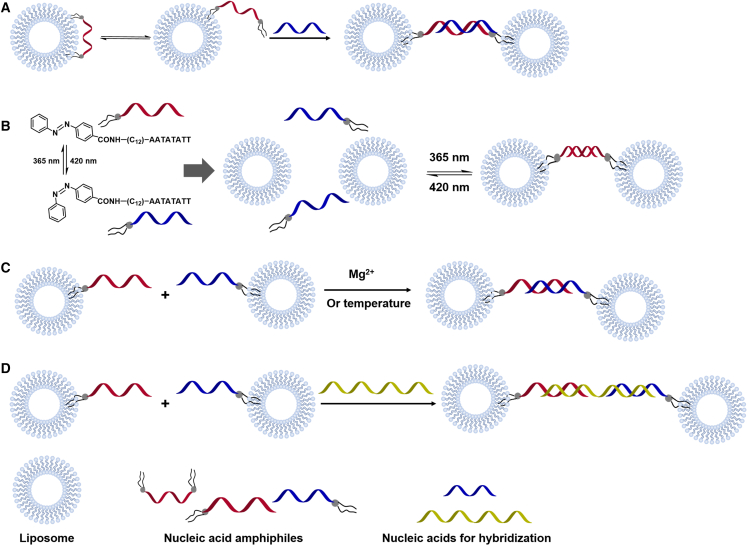
Liposome assembly mechanism induced by nucleic acid amphiphiles (A) After hybridization with a complementary strand, the increased stiffness from the formation of double-stranded nucleic acids no longer allows the two hydrophobic ends to anchor on the same liposome. (B) Light-responsive liposome assembly strategy by terminal pairing of nucleic acids with single hydrophobic modification. (C) Mg^2+^ or temperature-responsive liposome assembly strategy by terminal pairing of nucleic acids with single hydrophobic modification. (D) A third oligonucleotide that partially complements to nucleic acid amphiphiles is involved in inducing liposomes assembly.

Because nucleic acid-mediated liposome assembly has certain application potentials in the field of nucleic acid detection, Vogel’s group conducted research on nucleic acid-mediated liposome assembly in this field’s early days.^79^^,^^84^ Referring to an earlier report,^85^ they conjugated cholesteryl or palmityl moieties at both terminals of nucleic acid. The two ends of synthesized nucleic acid amphiphiles were reversibly anchored into the same liposome. After hybridization with a complementary strand, the increased stiffness of the formed double-stranded nucleic acids no longer allowed the two hydrophobic ends to be anchored to the same liposome. As a result, liposome assembly induced by nucleic acid hybridization was achieved (Figure 4A). Jakobsen et al. further found that nucleic acid amphiphiles with only one end able to leave the liposome surface are sufficient to induce liposome assembly.^86^ Using triple helix formation, they further achieved faster assembly of liposomes at lower concentration.^87^ Serien et al. used vitamin E-modified nucleic acid to achieve the aggregation of virus-like particles through the same mechanism,^82^ suggesting the potential applications of this assembly in related fields. Vogel’s group also explored the assembly strategy by a terminal pairing of nucleic acids with single hydrophobic modification; however, no liposome assembly was observed. The researchers believed that it was necessary to add a spacer between the nucleic acid and the hydrophobic modification to increase the distance between the nucleic acid and the liposome.^86^ Yet, Hernández-Ainsa and co-workers successfully used the same strategy (Figures 4B and 4C) to achieve reversible assembly of liposomes in response to light, Mg^2+^, and temperature.^88^^,^^89^ Dave and Liu established another assembly strategy: a third oligonucleotide that is partially complementary to nucleic acid amphiphiles was involved in inducing liposome assembly (Figure 4D). They meticulously studied the influence of liposome composition, the oligonucleotide linker and other factors on the properties of the assembly.^90^ Matsumoto et al. subsequently proved that Dave’s strategy is more efficient than that of Vogel for liposome assembly.^91^

**Figure 5:**
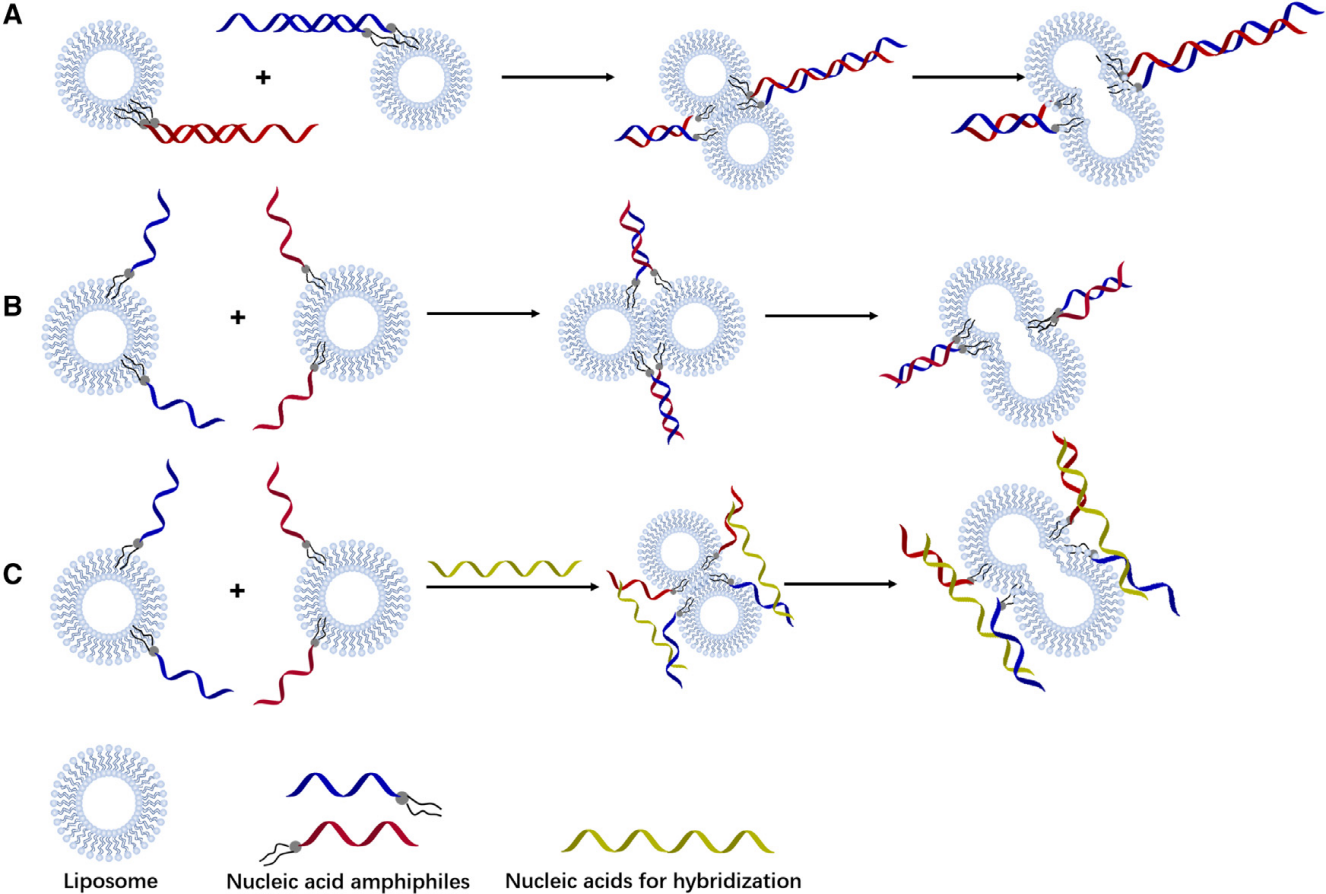
Nucleic acid amphiphile-mediated liposome fusion mechanism (A) Double-stranded nucleic acid amphiphiles hybridization-induced liposome fusion. (B) Single-stranded nucleic acid amphiphile hybridization-induced liposome fusion. (C) Double-zipper design for liposome fusion by a third nucleic acid.

Nucleic acid-mediated particle assembly is a topic that has been studied for a long time. Similar research on inorganic nanoparticle assembly was conducted considerably earlier and is more established than that of nucleic acid-mediated liposome assembly. Research on nucleic acid-mediated liposome fusions is more representative of the uniqueness of nucleic acid amphiphile-modified liposomes. Membrane fusion occurs in various important biological processes, such as cell endocytosis, exocytosis, and virus infection. This process generally consists of three steps: (1) the membranes approach each other, (2) the original membranes undergo deformation and rearrangement, and (3), the contents of the membranes mix to exchange their inner material.^92^ In eukaryotic cells, membrane fusions are mediated by membrane fusion proteins such as SNARE (soluble N-ethylmaleimide-sensitive factor attachment protein receptors).^93^ Nucleic acid amphiphiles, which can be anchored to liposomes through hydrophobic interactions, provide a simple way to mimic biological membrane fusions triggered by SNARE. The fusion efficiency and rate are influenced by multiple factors, such as the types of hydrophobic groups, the modification density of nucleic acid amphiphiles on the liposome, the sequence of nucleic acids, the linker between the nucleic acid and hydrophobic group, and the composition of the liposomes and solution environment. The key parameters for liposome fusions are the efficiency of content mixing and the degree of content leakage. By simulating the process of membrane fusions induced by SNARE zipper mechanism, Höök and co-workers first designed and explored liposome fusions induced by nucleic acid hybridization (Figure 5A).^72^^,^^94^ They further investigated the factors that affect membrane fusions with cholesterol-DNA amphiphiles.^94^ In their experiments, the increased density of nucleic acids on the liposome had no significant effect on the fusion efficiency. Simonsson et al. even found that liposomes modified with a high nucleic acid modification density have low fusion efficiency.^95^ In the same period, Boxer’s group designed another strategy.^96^^,^^97^ In their work, they modified nucleic acids with dual octadecane molecules. Dragged by two pairs of complementary oligonucleotides, the liposomes were forced into close proximity, which triggered subsequent fusion (Figure 5B). In contrast to Fredrik’s result, they observed that fusion efficiency was enhanced as the modification density of the nucleic acid increased.^96^ In addition, membrane fusion mediated by repetitive nucleic acid sequences such as poly(T) was more efficient. Moreover, a long non-complementary nucleic acid linker reduced the efficiency of membrane fusion owing to the inability to draw the liposomes close enough.^97^ Based on this strategy, Vogel’s group further investigated the factors that affect liposomes fusion, including different types of lipid molecules, nucleic acids (peptide nucleic acids, PNA), PEG linkers, and temperatures.^98^^,^^99^^,^^100^ Recently, Vogel’s group established a third strategy to program liposome fusions via a double-zipper design with remarkable efficiency (Figure 5C).^101^ Apart from terminal-modified amphiphiles, Meng et al. succeeded in using a hydrophobic group-modified nucleobase to construct nucleic acid amphiphiles and trigger liposome fusions efficiently.^102^

To summarize, nucleic acid amphiphile-induced liposome assembly and fusion have been thoroughly studied over the past two decades and considerable knowledge has been gained. Great achievements have been witnessed on the application of these models, such as microRNA detection by DNA-mediated liposome fusion^103^ and single-particle combinatorial liposome fusion mediated by DNA (SPARCLD) for the multiplexed cargo delivery of attoliter lipidic nanocontainers.^104^ However, there are still important potential uses of these models that are yet to be explored. Further research is warranted to fully understand the promising prospects of nucleic acid amphiphiles.

### Interactions between nucleic acid amphiphiles and the cell membrane

Understanding the interactions between nucleic acid amphiphiles and the cell membrane would be useful for their applications in cell membrane engineering and nucleic acid drugs delivery.^105^ The factors affecting the interaction of nucleic acid amphiphiles with the cell membrane can be summarized as environmental properties, physiochemical properties, and membrane properties. More detailed information can be found in Zhao’s review (Figure S4).^106^ The interaction process between the nucleic acid amphiphile and the cell membrane can be divided into three steps: adsorption, insertion, and endocytosis. It should be noted that these processes are dynamically reversible. Fetal bovine serum in the cell culture medium may also adsorb nucleic acid amphiphiles and cause unstable anchoring.^107^ For applications in cell membrane engineering, a long anchoring time for nucleic acid amphiphiles on the cell membrane is desirable. For this purpose, increasing the insertion efficiency and minimizing the endocytosis of nucleic acid amphiphiles are necessary.^108^

First, whether the nucleic acid amphiphiles can be stably anchored on the cell membrane is closely related to the hydrophobic group. In general, nucleic acid amphiphiles gain a higher insertion efficiency with greater hydrophobicity. Hydrophobicity can be regulated by different types and number of hydrophobic groups,^73^^,^^109^^,^^110^ or by altering the length of alkane chains.^107^ Summarized from current literature reports, the order of anchoring efficiency is: dual lipidic chains > cholesterol > vitamin E > single lipidic chain.^111^ However, when the hydrophobicity is too strong, the self-assembly of nucleic acid amphiphiles competes with membrane absorption and results in low insertion efficiency.^112^ To solve this problem, Weber et al. established a stepwise assembly method, in which a first anchor strand (Anch) is initially added and allowed to be anchored on the cell membrane. Then, a second, co-anchor (cA)strand with a sequence complementary to the sequence of Anch is added. The two strands hybridize with each other on the cell membrane and increase the total hydrophobicity of the nucleic acid amphiphiles. Thus, they can achieve stable anchoring and simultaneously decrease aggregation in the solution.^113^ Even so, this method is not applicable to anchoring nucleic acid nanostructures on the cell membrane, because more hydrophobic modifications are required for nucleic acid nanostructures. For this, it is not only necessary to ensure that the nanostructures do not aggregate in the solution, but also to ensure that they do not aggregate after being anchored to the membrane.^114^^,^^115^ Researchers have attempted to reduce aggregations of nucleic acid amphiphiles by decreasing the number of hydrophobic modifications, shortening the length of the lipidic chain, and altering the position of hydrophobic moiety on nucleic acid.^116^ The affinity toward the cell membrane was also decreased at the same time. Detailed research on the factors affecting nucleic acid amphiphile aggregation and cellular membrane anchoring has inspired the development of optimized structure. Ohmann et al. investigated the regulatory factors of cholesterol-nucleic acid aggregation, laying a foundation for the structural optimization of cholesterol-modified nucleic acids.^71^ Kurz et al. studied the interaction between vitamin E-modified nucleic acid and the cell membrane, and found that vitamin E-modified nucleic acid amphiphiles can be quickly adsorbed on the cell membrane and tend to accumulate in the disordered part of the liquid phase. This phenomenon indicates potential applications in drug delivery and colocalization with membrane proteins.^117^ In addition to direct insertion into the cell membrane, the lipid part of nucleic acid amphiphiles may also bind to proteins *in vivo* or in serum^118^ and interact with the cell membrane through receptor-mediated interactions. This type of interaction is generally accompanied by endocytosis and is mainly used in the field of drug delivery.

Linkers between nucleic acids and hydrophobic groups also have an effect on amphiphiles anchoring on cell membrane.^119^ Therefore, the use of linkers must also be carefully determined according to the specific applications. Although hydrophilic linkers, such as PEG, decrease insertion efficiency.^110^ They can inhibit endocytosis to some extent.^120^^,^^121^ Hydrophobic linkers can increase the insertion efficiency of the nucleic acid amphiphiles to a certain extent. However, if the linker is too long, a decreased insertion efficiency is also observed. It will also affect the intracellular transport of the nucleic acid amphiphiles after endocytosis.^119^ In addition, the presence of linkers can minimize the impact of cell membranes on the properties of nucleic acids including hybridization and target recognition properties.^122^

The length of the nucleic acid chain also has a profound impact on the membrane insertion efficiency of nucleic acid amphiphiles. The insertion efficiency of nucleic acid amphiphiles into lipid membranes decreased with longer nucleic acid segments. This can be attributed to the increased hydrodynamic pull of the fully hydrated negatively charged backbone, which increases steadily with length extension. Liu et al. held the opinion that nucleic acid amphiphiles with long nucleic acid chains form micelles with greater charge density,^11^ which reduces the accessibility of lipid molecules to the cell membrane, whereas Palte and Raines considered that the negatively charged glycocalyx will cause the electrostatic repulsion of nucleic acids.^107^ In addition to the structure of the nucleic acid itself, the concentration of the nucleic acid amphiphile also affects the insertion efficiency. Obviously, we can anchor a greater density of nucleic acids onto the cell membrane with a higher initial concentration,^108^^,^^123^ however, an excessive anchoring density may adversely affect the hybridization ability of the nucleic acids.

After the nucleic acid amphiphile is anchored to the cell membrane, it may be endocytosed into the cells through the endocytosis pathway.^124^ However, it can then be discharged through the circulation of a vesicle, making it unable to anchor onto the membrane stably for a long time. Therefore, the inhibition of endocytosis is also an important subject when studying the interaction between nucleic acid amphiphiles and cell membranes. Li et al. reported that modification of three cholesterols on the triangle of nucleic acid tetrahedra can effectively inhibit endocytosis, which may be related to the larger negatively charged structure of the nucleic acid tetrahedron.^109^ In addition, endocytosis can be inhibited by adding endocytosis inhibitors.^112^

As expected, the structures of nucleic acid amphiphiles play a vital role in determining their properties. When designing a nucleic acid amphiphile for a specific application, its properties are predicted through rational design based on the knowledge gained from previous basic research. At the same time, experimental data can help advance our understandings of the refined framework of the structure-properties relationship for nucleic acid amphiphiles.

## Applications of nucleic acid amphiphiles

The research discussed above on the synthesis and properties of nucleic acid amphiphiles lay the foundation for their further biological applications. Self-assembly of nucleic acid amphiphiles, nucleic acid amphiphile-modified liposomes, and single nucleic acid amphiphiles have found applications in drug delivery. Nucleic acid amphiphiles and nanostructures formed from nucleic acid amphiphiles with the ability to anchor stably onto cell membranes have been applied in cell membrane engineering and analysis. Recently, the mechanism of nucleic acid amphiphile-induced liposome fusion was applied for microRNA detection. These applications are discussed in detail in this section.

### Nucleic acid amphiphiles for drug development

Coupling highly hydrophobic molecules to nucleic acid drugs may improve delivery efficiency into cells. Taking hydrophobic siRNA as an example, chol-siRNA, known as the famous “cholesterol conjugated siRNA,” has been studied for decades. Terminal modification of cholesterol through the *trans*-4-hydroxyproinol linker^14^^,^^125^^,^^126^ or TEG linker^127^^,^^128^ can enable it to anchor to cell membranes (Figure S5A). After internalization by the cell and escape from the endosome, the siRNA is released, triggering RNAi effect. However, only up to 50% of the RNAi effect may be obtained, even at an extremely high concentration of chol-siRNA,^119^^,^^129^ which is mainly due to low endosomal escape efficiency (usually <0.01%).^130^ Considerable effort has been made to solve or avoid siRNA endosomal trapping^131^^,^^132^^,^^133^; however, limited improvements have been reported. Notably, cholesterol can bind to albumin (K_d_ ∼1 μM), which extends the serum half-life of siRNA from <10 to ∼90 min.^125^ Another hydrophobic moiety-vitamin E is also commonly used in nucleic acid drug modification with enhanced cell penetration, increased endogenous gene inhibition, and improved pharmacokinetics.^134^^,^^135^^,^^136^ Fatty acids such as palmitic acid have also been conjugated to the 5′ terminal of antisense oligonucleotides to treat myelofibrosis.^137^^,^^138^^,^^139^ In addition to its cell penetration ability, fatty acids also bind to albumin. Thus, a prolonged serum half-life of nucleic acid drugs can be obtained. However, fatty acids can also bind to fatty acid-binding proteins, which are widely distributed in various tissues of the body, resulting in trapping of the drugs in those tissues and then the decrease of drug in blood circulation.^140^ A recent report by Kauss et al. verified the efficient uptake of alkane-modified oligonucleotides by prokaryotic cells for the first time.^141^ More detailed discussion on this topic can be found in several recent reviews.^142^^,^^143^

Apart from the delivery of nucleic acid drugs, the micelles formed by nucleic acid amphiphiles are also used in the delivery of poorly water-soluble drugs (Figure S5B).^144^^,^^145^ In addition to self-assembly, nucleic acid amphiphiles are also applied to endow other drug carriers with better delivery performances. A commonly used strategy is to anchor complementary nucleic acids onto cell membranes and liposomes, to achieve targeted delivery through nucleic acid hybridization.^146^^,^^147^ Furthermore, since parts of nucleic acids such as aptamers have targeting capabilities to cells themselves, direct modification of these nucleic acids on drug carriers can also achieve specific cell targeting. For example, Yerneni et al. anchored the cholesterol-modified AS1411 aptamer to exosomes and successfully achieved the targeted delivery of exosomes to tumor cells. This is a simple and efficient method for exosomes modification.^148^ Moreover, the active groups can be modified at the other end of the nucleic acid amphiphiles to realize carrier modification by a variety of ligands thus endowing the drug carrier with the ability to target different cells.

In addition to improving delivery efficiency, conjugating nucleic acid drugs with highly hydrophobic groups might also improve the activity of the modified nucleic acid drug. Researchers such as Xodo and co-workers have explored the impact of polycyclic aromatic hydrocarbon units (especially, nucleic acid intercalator) on the folding, stability, and potency of triplex-forming oligonucleotides^149^^,^^150^^,^^151^ and G-quadruplex.^152^^,^^153^^,^^154^ In these cases, the hydrophobic modifications improve the activity of the corresponding nucleic acid drugs mainly through thermal stabilization of the functional structure and improvement of nuclease resistance. Another interesting example of the impact of hydrophobic modifications on the activity of nucleic acid drugs is the development of the modified Hotoda’s sequence as an anti-HIV agent.^155^ In this case, the hydrophobic group can arrange the formed G-quadruplex structure and improving the recognition toward the target (Figure S5C).

Collectively, nucleic acid amphiphiles have the potential to improve the delivery efficiency of nucleic acid drugs and small-molecule drugs. In addition, hydrophobic moieties can participate in the structure formation and target recognition of nucleic acid amphiphiles. In addition to being used in nucleic acid delivery systems, nucleic acid amphiphiles have been used for small-molecule delivery or enhancing the activity of nucleic acid drugs, though its application is still in infancy.^156^ It is worthy of exploring the impact of hydrophobic groups on the structure folding and target recognition of aptamers in the future.

### Nucleic acid amphiphiles in cell membrane engineering

Under specific conditions, nucleic acid amphiphiles can anchor to cell membranes. Benefiting from diversity and programmability of nucleic acid structures, many applications related to cell membranes have become possible, including modeling cell-to-cell connections, simulating the function of transmembrane proteins using nucleic acid nanostructures, and analyzing biological events near the cell membranes. Compared with covalent modification of cell membranes and functionalizing cell membranes through genetic engineering, anchoring of nucleic acid amphiphiles to cell membranes is a simpler and harmless method. Such cell membrane engineering approaches cannot only endow cells with new functions and make new cell therapies possible but also deepen people’s understanding of related biological issues through a bottom-up process.

**Figure 6:**
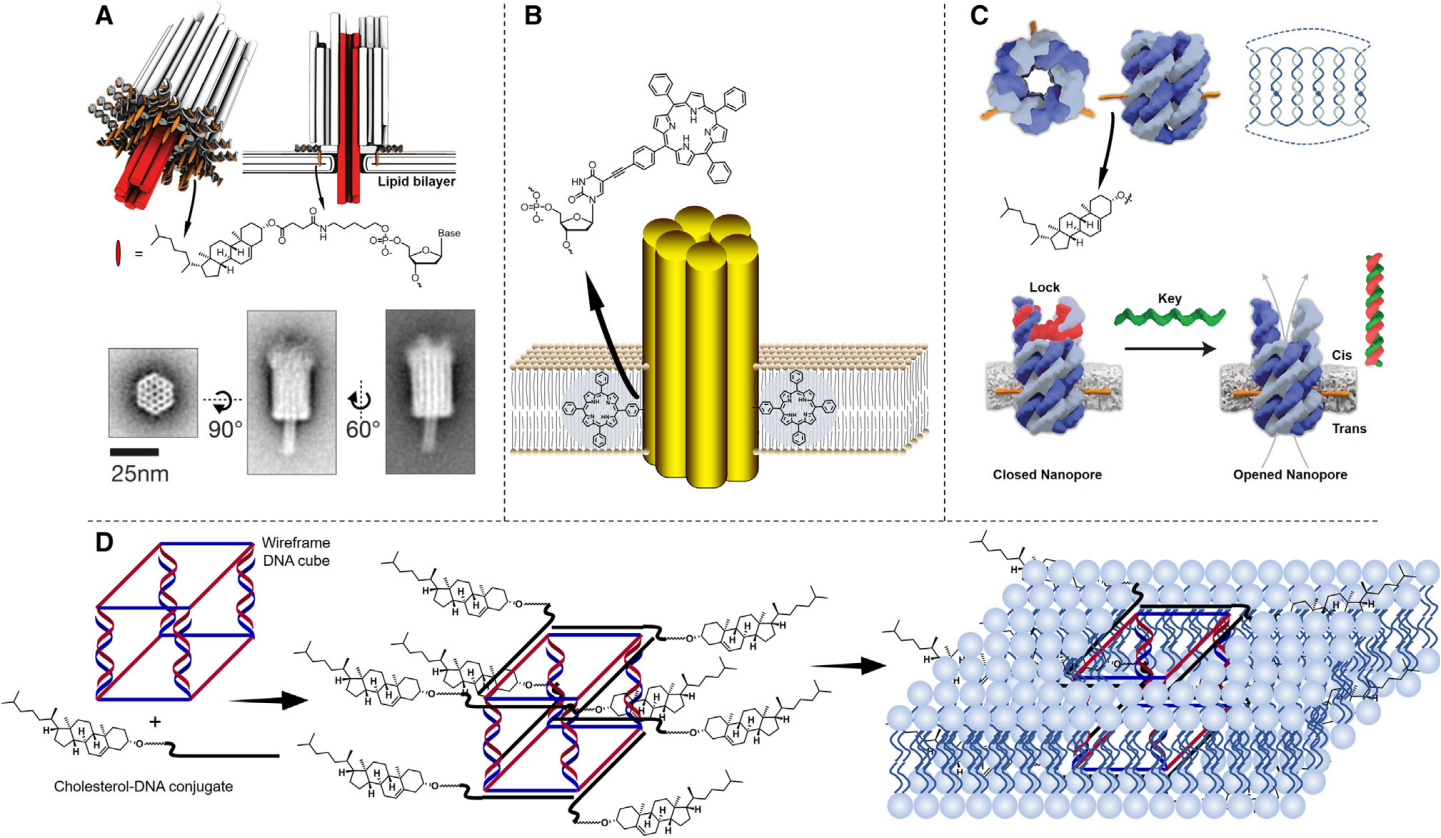
Synthetic DNA membrane channels (A) DNA channels adhering to small unilamellar vesicles (SUVs) made from POPC (1-palmitoyl-2-oleoyl-sn-glycero-3-phosphocholine) lipids (adapted from Langecker et al.^158^). (B) A DNA nanopore composed of six interconnected duplexes and carrying porphyrin-based lipid anchors (adapted from Burns et al.^159^). (C) The channel opening of DNA nanopore using “key” DNA to remove the “lock” DNA (adapted from Burns et al.^160^). (D) Spatial presentation of cholesterol units on a DNA cube as a determinant of membrane protein-mimicking functions (adapted from Chidchob et al.^161^).

An important research area of cell engineering is the use of nucleic acid amphiphiles (mainly composed of nanostructures conjugated with lipidic molecules) to simulate the functions of membrane proteins. The nucleic acid nanopores can be used to simulate the transport channels onto the cell membrane. Typically, these transmembrane channels consist of membrane proteins, which facilitate the transport of water, ions, and other entities. The significance of simulating the transmembrane channel functions with nucleic acid nanostructures is that the size and shape of the nanopores can be tuned more conveniently to meet specific requirement.^157^ Nucleic acid nanopores are extremely large, rigid, and negatively charged structures. It is conceivable how much of the energy barrier must be overcome to insert them into the cell membrane. In early reports, researchers conducted modification using 26 cholesterols on nucleic acid nanopores for their insertion into the cell membrane (Figure 6A),^158^ and 57 vitamin E molecules were involved in another study.^162^ Subsequently, researchers introduced hydrophobicity via an ethyl phosphorothioate-modified scaffold to remove negative charges on the phosphate oxygen.^163^^,^^164^ However, this method needs to change multiple sites in the nucleic acids, which is extremely cumbersome. Later, they turned to tetraphenylporphyrin with superior hydrophobicity and found that only two modifications with such large hydrophobic groups were needed on the nucleic acid nanostructure to anchor it onto the cell membrane (Figure 6B).^159^^,^^165^ In addition, simplifying the nucleic acid nanopores can also reduce the number of required hydrophobic group modifications (Figure 6C).^160^ To date, the smallest biomimetic nucleic acid nano ion channel is composed of only a simple double-strand DNA structure.^166^ Burns et al. conducted a comprehensive study on the interaction between cholesterol-modified nucleic acid nanopore and bilayer membrane. They investigated the process of a nucleic acid nanopore, modified with three cholesterols, interacting with the bilayer membrane from absorption to insertion. They simultaneously explored the relationship between insertion efficiency and cell membrane curvature,^167^ and the endocytosis of the cell membrane to these structures was also studied.^168^ Recently, Chidchob et al. designed a novel cube structure whose upper and lower sides are composed of single-stranded nucleic acids. Cholesterol can be incorporated onto the corners of the cube via base pairing with oligonucleotides modified with cholesterol. Interestingly, owing to the particularity of this structure, nucleic acid cubes modified with cholesterol at different corners undergo completely different interactions with the membrane, giving this structure a wide range of application prospects (Figure 6D).^161^

**Figure 7:**
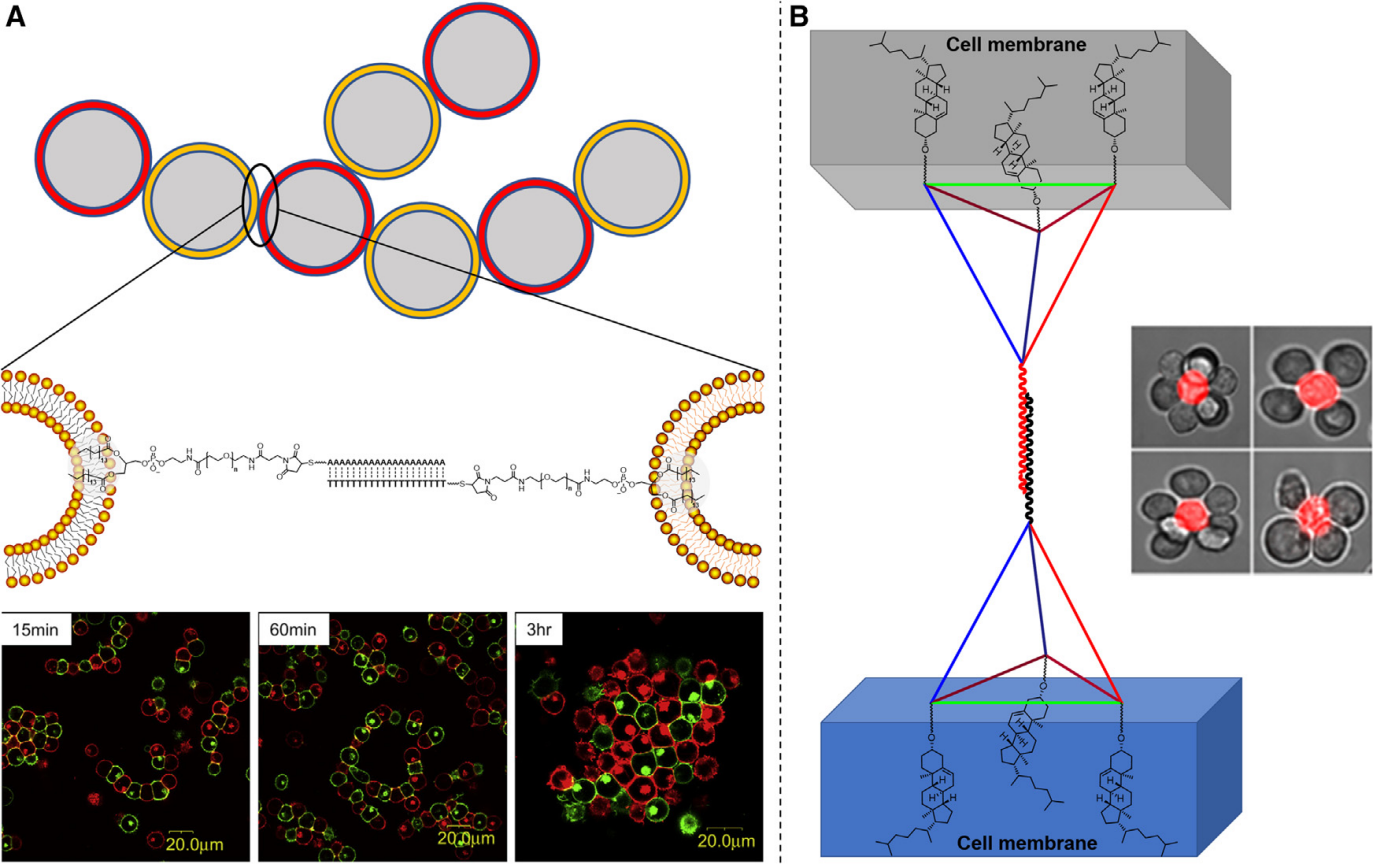
Cell to cell attachment mediated by nucleic acid amphiphiles (A) Cell attachment through nucleic acid amphiphiles incorporation and poly(T)-poly(A) hybridization (adapted from Teramura et al.^120^). (B) Cellular assembly by DNA tetrahedral amphiphiles (adapted from Li et al.^109^).

Another important application in cell membrane engineering is the rebuilding of intercellular interactions. For nucleic acid amphiphiles, either the hybridization properties of nucleic acids or specific structures such as aptamers are utilized to simulate a series of biological processes such as interactions between cells and adhesion of cells to matrix. The rebuilding of intercellular interactions is the foundation of tissue rebuilding.^169^ In these applications, nucleic acid amphiphiles exhibit great programmable capability and convenience of synthesis and chemical modification.^170^ The incorporation of nucleic acid amphiphiles into cell membranes, as mentioned above, offers negligible cell toxicity and convenient incorporation process. Borisenko et al. first reported an investigation that used nucleic acid amphiphiles anchored on cell membrane to simulate intercellular connections and adhesion of cells to matrix. In their study, they mentioned the possible problems that must be overcome, which required adequate anchoring stability of nucleic acid amphiphiles onto a cell membrane with little endocytosis, and little dissociation from the cell membrane.^108^ Teramura et al. performed a similar investigation in the same period. In contrast, they added a long PEG linker between the nucleic acid and lipidic molecules. Although they were not mentioned in their paper, from their fluorescent images, we could observe endocytosis of nucleic acid amphiphiles and their aggregation in the cell with prolonged incubation time (Figure 7A).^120^ However, nucleic acid amphiphiles have still been successfully applied to intercellular interaction-related biological events, such as cell invasion,^171^ signal transmission between taste cells and nerve cells,^172^ and cell-to-cell adhesion.^173^ Compared with other covalent cell membrane modification methods, Nicholas Selden et al. claimed that nucleic acid amphiphiles have a better modification efficiency. During their evaluation of cell membrane modification efficiencies with different lipidic molecules, they observed that dual C_16_ alkane-nucleic acids had the highest incorporation efficiency.^170^ A similar result for the influence of lipids on the anchoring efficiency of nucleic acid was obtained by Tokunaga et al.^174^ And more detailed discussion can be found in the research paper by Weber et al.^113^ In addition, Selden et al. found that a long linker between nucleic acids and lipidic molecules benefits base pairing between nucleic acids by decreasing steric hindrance induced by glycocalyx on the cell surface. Recently, Li et al. introduced nucleic acid amphiphiles with tetrahedral structure to construct cell connections. This structure could be more stably anchored on to the cell membrane (neither endocytosis nor shedding was observed) than previously reported structures and improve the performance of this artificially constructed cell connection to some extent, making the study of related biological events more precise (Figure 7B).^109^ In addition to base pairing, aptamers recognizing corresponding receptors on the cell surface are also used for cell membrane modification to achieve special functions. With aptamer amphiphiles, Xiong et al. modified cancer cell-targeting aptamers on immune cells, so that the immune cells could target cancer cells more specifically, and improved the effectiveness of cellular immunotherapy in killing tumor cells.^122^

### Nucleic acid amphiphiles for bioanalysis

**Figure 8:**
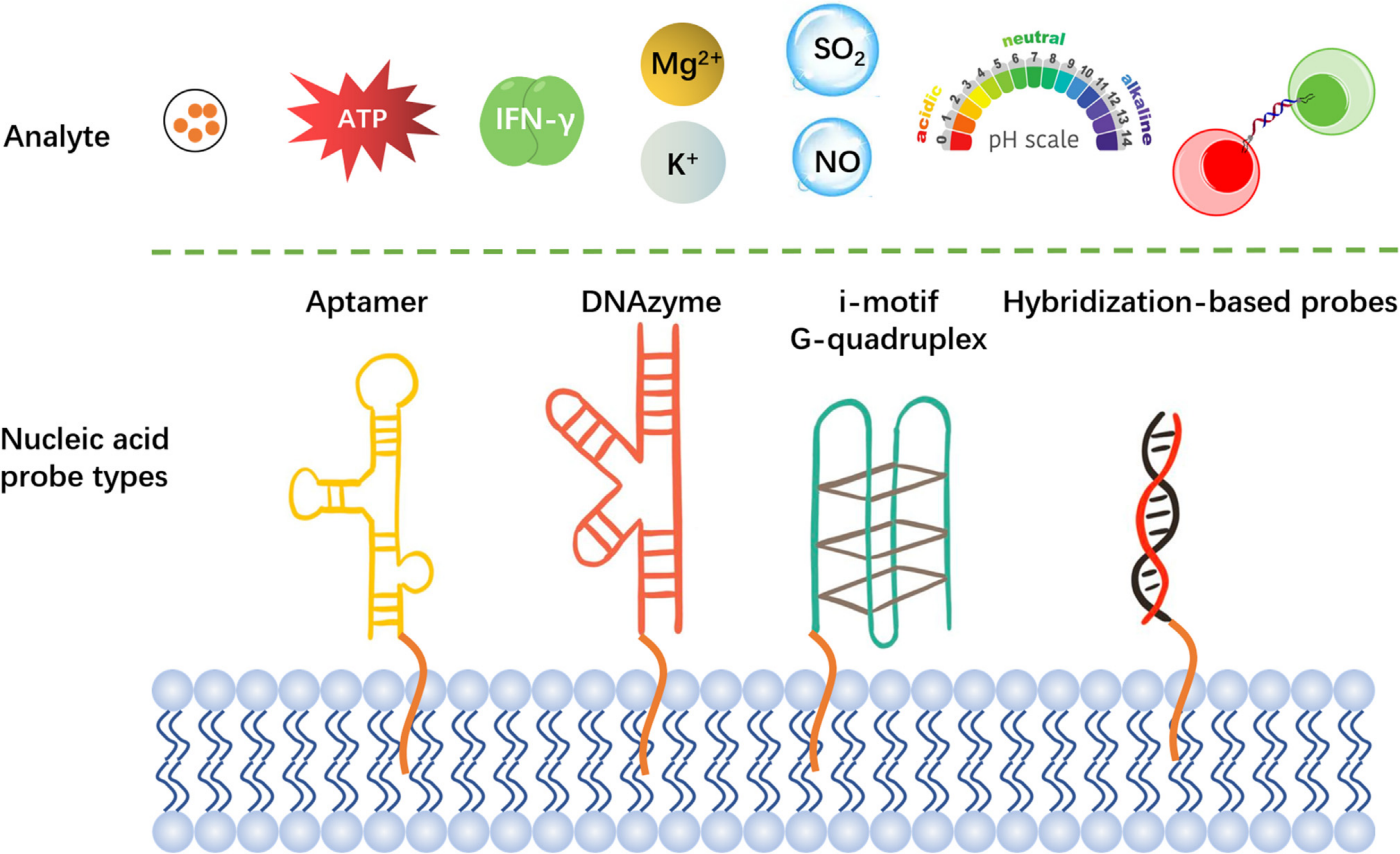
Nucleic acid amphiphiles with different nucleic acid probe types for bioanalysis of different analytes

Cell membrane plays vital roles in biochemical events. The detection of microenvironmental changes near/on the cell membrane is of great significance to the understanding of related biochemical events. Nucleic acid amphiphiles that can be anchored to the cell membrane, with a combination of specific anchoring properties of the lipid part and the high functional diversity of the nucleic acids, have been developed for the detection of a variety of signal substances in the cell membrane microenvironment (Figure 8), such as adenosine triphosphate (ATP), metal ions, gas molecules, and neurotransmitter. In the past few decades, researchers have screened a large number of aptamers that bind to small molecules. And these aptamers are widely applied to biosensing. Up to now, aptamers modified with lipidic molecules have been utilized to detect neurotransmitters,^121^ ATP,^175^ and interferon gamma.^176^ Apart from aptamers, several other nucleic acid structures are responsive to metal ions. For example, ribozymes can cleave the target nucleic acid in the presence of metal ions. Several quadruplexes can only be formed in the presence of metal ions. These special types of nucleic acids can also be used in biological analysis. Tan’s group successively took advantage of the DNAzyme^121^ and quadruplex^177^ that anchor onto the cell membrane to detect Mg^2+^ and K^+^ near the cell membrane. Gas molecules are also a common type of signaling molecules. However, so far, gas-responsive nucleic acid molecules have not been reported. Interestingly, Feng et al. constructed a small molecule that could react with HSO_3_^−^ through Michael addition. This molecule produces strong fluorescence upon binding to the G-quadruplex, and the fluorescence intensity decreases after reacting with HSO_3_^−^. Thus, the researchers obtained a nucleic acid-based SO_2_ biosensor. Similarly, they constructed another G-quadruplex-based NO biosensor using the same design principle. These cell membrane-anchored gas molecule responses enable real-time monitoring of gas signal transmission.^178^

The extracellular pH is related to a variety of physiological and pathological processes, hence the detection of extracellular pH has always been a research hotspot. Ke et al. firstly tried to apply nucleic acid amphiphiles to construct ratiometric fluorescent probes for the detection of extracellular pH. In their study, the hydrophilic nucleic acids of amphiphiles were merely regarded as a linker between the lipidic part that anchored in the cell membrane and the pH-responsive fluorescent molecules, which guaranteed that the pH-responsive moieties would protrude from the cell.^179^ Based on the early discovery that cholesterol-modified DNA tetrahedrons can be stably anchored onto cell membranes,^109^ Liu et al. constructed a ratio metric fluorescent probe using pH-responsive DNA tetrahedrons modified with cholesterols and applied it to monitor the pH changes in synaptic vesicles.^180^ The nucleic acids did not play a part in the imaging process in this study either. The cases that truly utilize nucleic acids as pH-responsive moieties include the formation of i-motif structures, which are composed of two parallel DNA duplexes rich in cytosine that occur at low pH values with the protonation of cytosine but unwind at high pH values with the deprotonation of cytosine.^67^ Zeng et al. first designed a DNA zipper probe based on the i-motif structure and fluorescence resonance energy transfer mechanism to detect pH changes outside the cell.^181^ However, their design has two flaws. First, the use of the i-motif itself only allowed a narrow pH response range and, second, only one cholesterol was added in their design for anchoring onto the cell. As previously mentioned, it is challenging to stably anchor onto the membrane with only one cholesterol modification.^73^ Recently, Liu et al. reported a pH probe based on a nucleic acid triplex nanozipper.^182^ This probe was modified with three cholesterols to ensure that it can be anchored stably onto the cell membrane. The formation of a nucleic acid triplex depends on protonated cytosine. Thus, the pH response range can be expanded by adjusting the corresponding nucleic acid sequence in the probe.^183^

In addition to detecting changes in the microenvironment near the cell membrane, nucleic acid amphiphiles are also used to study the cell membrane itself.^123^ For example, Sun et al. used cholesterol-modified nucleic acids to study the distribution of the lipid rafts of cell membranes,^184^ and Zhao et al. used cholesterol-modified nucleic acids to study the mechanics of cell interactions.^185^ Similarly, You et al. successfully imaged transient interactions in live cell membranes by using cholesterol-modified programmable DNA probes known as “DNA Zippers.” Then, they measured the membrane order during the activation of T cell receptor signaling.^186^ Later, they continued reporting several novel DNA zippers, including sphingomyelin-conjugated DNA zippers that were specifically located in and detected membrane lipid-ordered domains, as well as a tocopherol-DNA zipper, which was used for the selective imaging of lipid-disordered phases.^187^

## Conclusions and perspective

During the past two decades, nucleic acid amphiphiles have been successfully applied to construct various nanostructures and have achieved a variety of applications in biomedicine and bioanalysis. In this paper, we review the solid-phase and solution-phase synthetic strategies that have been reported for the synthesis of nucleic acid amphiphiles. We then discuss the properties of nucleic acid amphiphiles in various contexts, including self-assembly properties, and interactions with liposomes and cell membranes. Finally, we explore the applications of nucleic acid amphiphiles in drug development, cell membrane engineering, and bioanalysis. Next, we propose several directions for this research area. Despite the diverse nucleic acid amphiphiles utilized to date, a reliable large-scale synthesis method for biomedical applications is still lacking. Solution-phase synthesis is generally considered more suitable for preparing nucleic acid amphiphiles on a larger scale. However, due to the difference in molecular polarity between nucleic acids and hydrophobic ingredient, it is quite challenging to remove the excessive hydrophobic ingredients completely, which often encase the target nucleic acid amphiphiles during the concentration process and decreased final yield of nucleic acid amphiphiles. Therefore, it is essential to explore novel synthetic strategies for large scales, which would benefit the transformation of nucleic acid amphiphiles from being limited to basic research to practical application. To study the properties of nucleic acid amphiphiles, it is critical for researchers to bridge the gap between the research and specific applications. Taking the relationship between nucleic acid amphiphiles and membranes as an example, the considerations vary with different applications. For membrane protein simulation, researchers pay more attention to the anchoring stability of the nanostructures formed by nucleic acid amphiphiles, while for drug delivery, researchers pay more attention to the transmembrane transport of nucleic acid amphiphiles. Thus, the structure-property relationship gained from research could guide the rational design of nucleic acid amphiphiles for specific applications. Furthermore, the properties of nucleic acid amphiphile observed during practice for specific applications would further expand our understanding of the rules behind nucleic acid amphiphile self-assembly and interactions with membranes. Currently, there is no standardized method to predict the properties of a nucleic acid amphiphile with a specific structure. More research with varied nucleic acids, hydrophobic moieties, and linkers is still needed to expand our understandings. After sufficient data have been collected, it may be possible to predict the properties of newly emerged nucleic acid amphiphiles using machine learning methods in the near future.

Finally, most research on nucleic acid amphiphiles focus on self-assembly, anchoring to lipid surfaces, and oligonucleotide delivery. However, the interactions between nucleic acid amphiphiles and biomacromolecules are rarely reported, except in several of the cases reported in this review, which deserve further study. Anyway, it is foreseeable that, as our understanding of nucleic acid amphiphile properties deepens in the future, the application scope of nucleic acid amphiphiles will greatly expand.

## Supplemental information

Document S1. Figures S1–S5

Document S2. Article plus supplemental information
