# Supplementary material for: Nucleic acid amphiphiles: Synthesis, properties, and applications
Source: Mol Ther Nucleic Acids. 2023 Jun 3;33:144–63. doi: 10.1016/j.omtn.2023.05.022 (PMC10345231; doi:10.1016/j.omtn.2023.05.022)
Supplement: Document S1. Figures S1–S5 [file mmc1.pdf]

OMTN, Volume *33*

Supplemental information

Nucleic acid amphiphiles: Synthesis, properties, and applications

Amu Gubu, Xueli Zhang, Aiping Lu, Baoting Zhang, Yuan Ma, and Ge Zhang

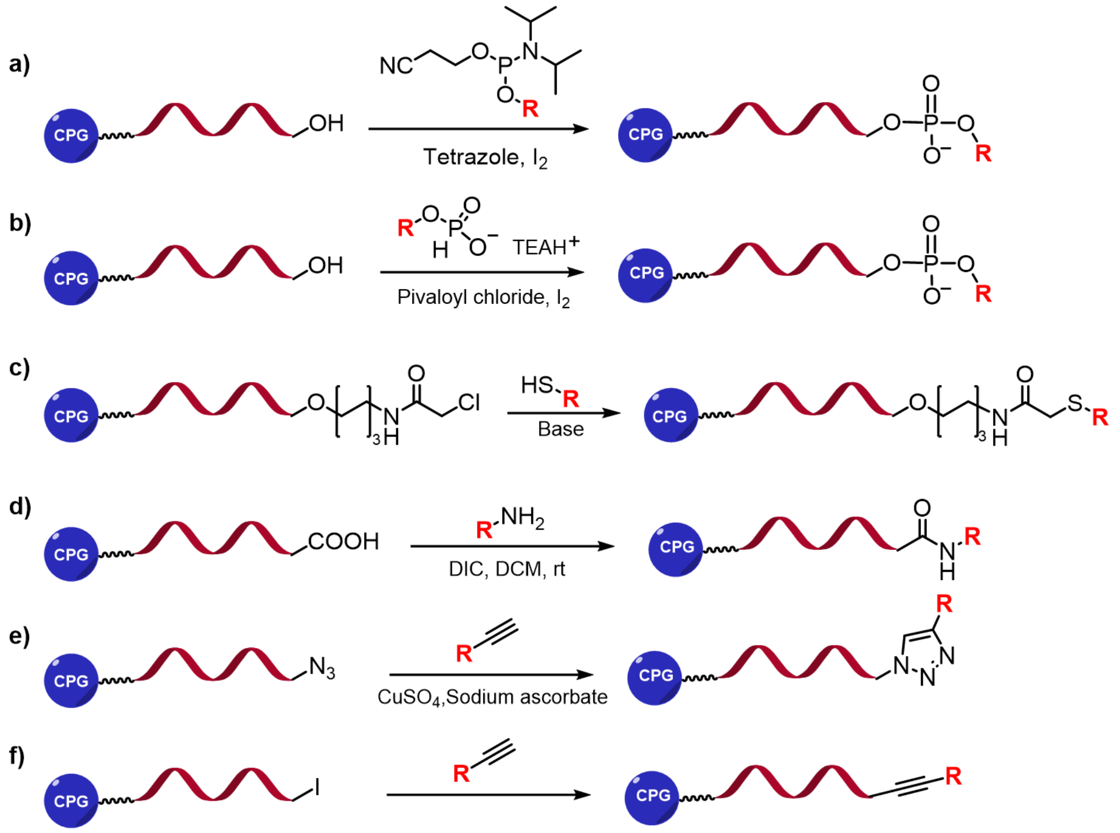

Figure S1. Solid-phase modification method for the synthesis of 5′ terminal-functionalized nucleic acid amphiphiles.

a) Phosphoramidite chemistry. b) H-phosphonate chemistry.[1] c) Substitution reaction between thiol and 2-chloroacetamide.[2] d) Amide formation reaction.[3] e) Copper catalyzed alkyne-azide cycloaddition.[4] f) Sonogashira coupling.[5] Red R represents hydrophobic moieties. CPG: Controlled Pore Glass, a high silica glass that includes pores between 50 and 300 nm which was excellently suited for the synthesis of oligonucleotides as the solid support.

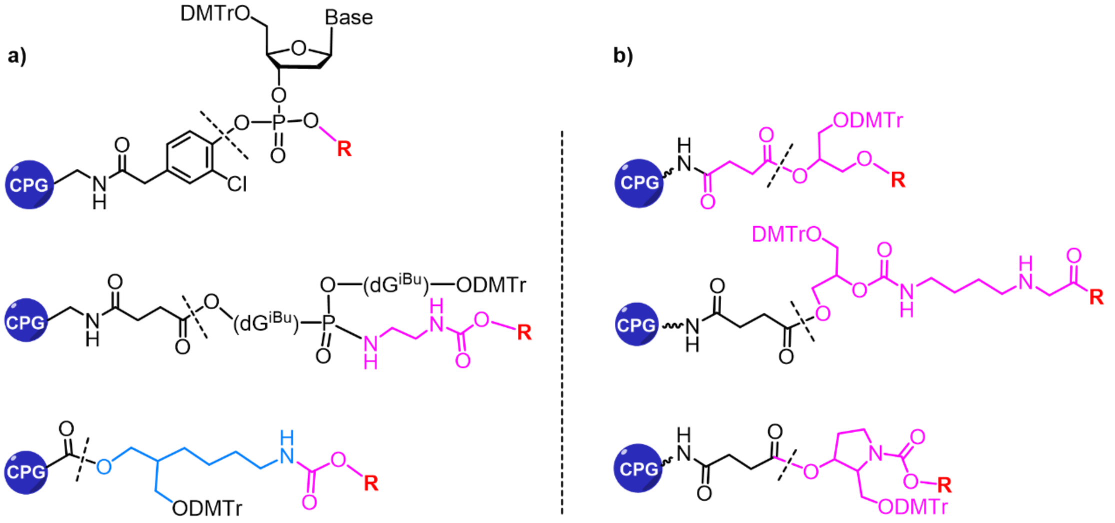

Figure S2. Strategies of hydrophobic modifications on the solid support at the 3′ terminus.

a) Hydrophobic groups were coupled to solid supports that extended linkers for coupling. b) Hydrophobic group-ligated linkers were directly conjugated to solid supports. DMTr represents 4,4'-Dimethoxytriphenylmethyl, a 5′-hydroxyl protecting group commonly used in DNA/RNA solid phase synthesis. Red R represents hydrophobic moieties. Purple-colored elements represent hydrophobic group-conjugated linkers. The dotted line represents the cleavage site after deprotection.

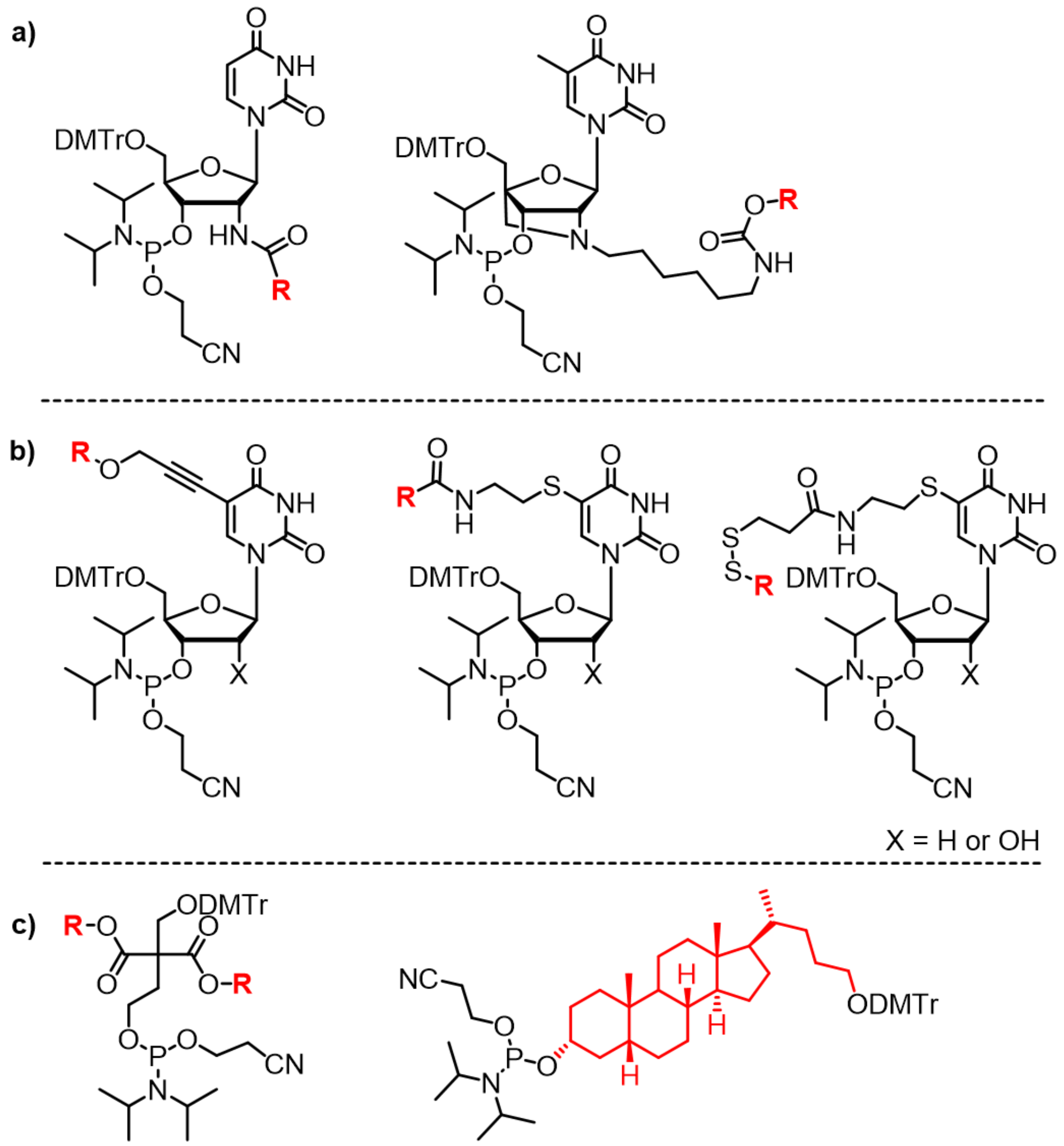

Figure S3. Phosphoramidites for synthesis of internal-functionalized nucleic acid amphiphiles.

a) Nucleoside phosphoramidites with hydrophobic modification at the 2′ position. b) Nucleoside phosphoramidites with hydrophobic modification at the 5′ position of thymidine or uridine. c) Non-nucleoside phosphoramidites for hydrophobic modification. The structure in red on the right is a lithocholic acid which is utilized as the hydrophobic moiety itself. Red R represents hydrophobic moieties.

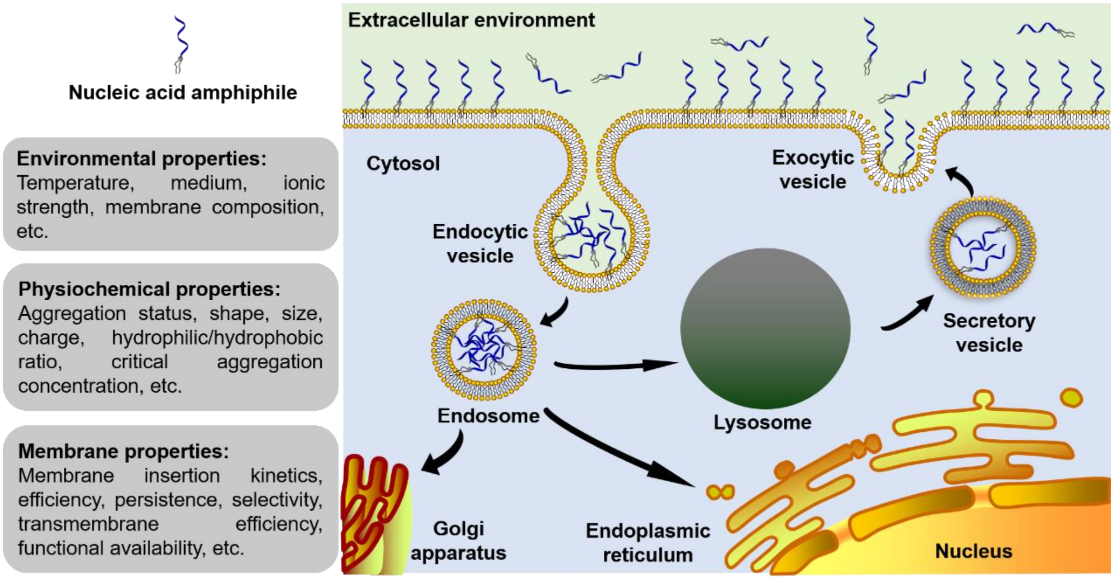

Figure S4. The factors influencing interactions between nucleic acid amphiphiles and the cell membrane (adapted from Zhao Bin, et al.).[6]

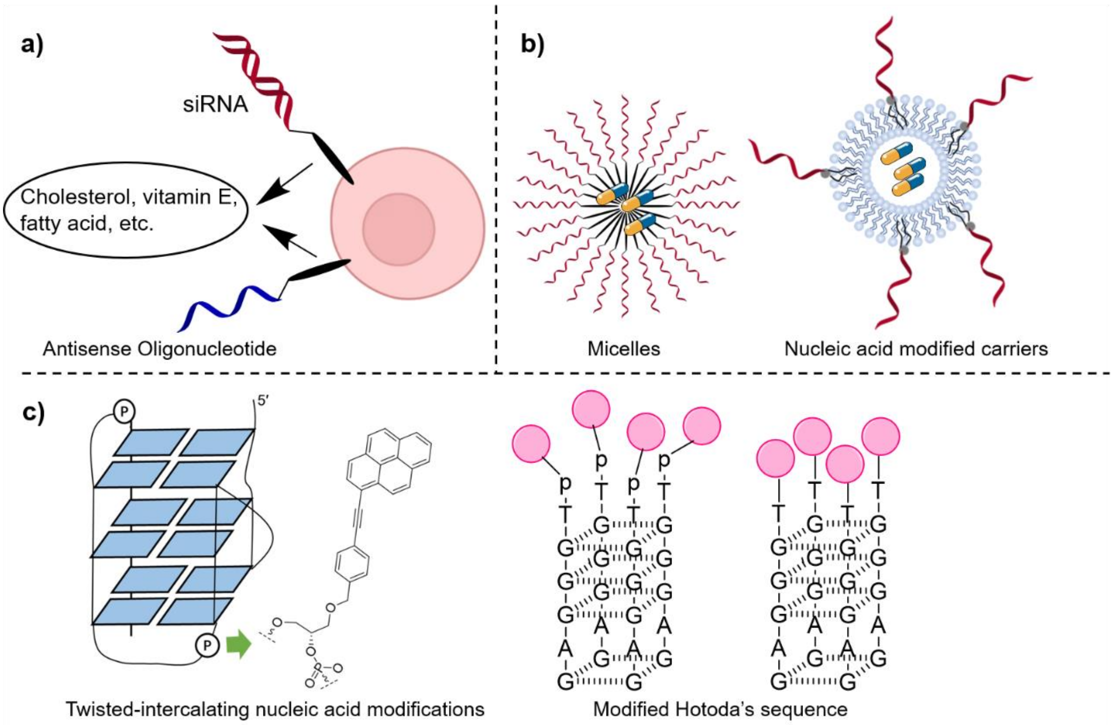

Figure S5. Nucleic acid amphiphiles for drug delivery and activity improvement. a) Nucleic acid amphiphiles for nucleic acid drugs delivery; b) Nucleic acid amphiphiles for small molecule drugs delivery; c) Nucleic acid amphiphiles for improvement of nucleic acid drug activity. Pink ball represents hydrophobic groups.

Reference

1. Shea, R. G., Marsters, J. C., and Bischofberger, N. (1990). Synthesis, hybridization properties and antiviral activity of lipid-oligodeoxynucleotide conjugates. *Nucleic Acids Res* **18**: 3777-3783.
2. Guzaev, A., and Manoharan, M. (1998). Conjugation of oligonucleotides via an electrophilic tether: N-chloroacetamidohexyl phosphoramidite reagent. *Biorg Med Chem Lett* **8**: 3671-3676.
3. Teixeira Jr, F., Rigler, P., and Vebert-Nardin, C. (2007). Nucleo-copolymers: Oligonucleotide-based amphiphilic diblock copolymers. *Chem Commun* **11**: 1130-1132.
4. Godeau, G., Arnion, H., Brun, C., Staedel, C., and Barthélémy, P. (2010). Fluorocarbon oligonucleotide conjugates for nucleic acids delivery. *MedChemComm* **1**: 76-78.
5. Yang, C. J., Pinto, M., Schanze, K., and Tan, W. (2005). Direct synthesis of an oligonucleotide–poly(phenylene ethynylene) conjugate with a precise one-to-one molecular ratio. *Angew Chem Int Ed* **44**: 2572-2576.
6. Zhao, B., Tian, Q., Bagheri, Y., and You, M. (2020). Lipid–oligonucleotide conjugates for simple and efficient cell membrane engineering and bioanalysis. *Curr Opin Biomed Eng* **13**: 76-83.
